# Immunomodulatory potential of dexmedetomidine in perioperative pain management for patients with cancer

**DOI:** 10.1016/j.cpt.2024.12.007

**Published:** 2025-01-02

**Authors:** Yuxian Liu, He Ma, Xintong Wang, Isabelle Yang, Jingping Wang

**Affiliations:** a Phillips Academy, Andover, MA 01810, USA; b Department of Anesthesia, The Second Hospital of Jilin University, Changchun, Jilin 130021 China; c Department of Anesthesia, Shenzhen Children's Hospital, Shenzhen, Guangdong 518172, China; d Department of Anesthesia, Critical Care and Pain Management, Massachusetts General Hospital, Harvard Medical School, Boston, MA 02114, USA

## Abstract

• Dexmedetomidine (DEX) has immunomodulatory effects in perioperative pain management.
• DEX can reduce pro-inflammatory cytokines.
• DEX has dose-dependent effects on tumor progression.
• Optimizing DEX dosing may improve the therapeutic outcomes of cancer surgery.
• Further research is needed to clarify DEX's impact on cancer recurrence and survival, as the findings remain mixed.

## Introduction

With growing concerns about the opioid crisis, the adoption of multimodal analgesia has significantly increased in clinical settings over the last 20 years. Multimodal analgesia refers to the use of various analgesic agents that act via distinct mechanisms at multiple sites along the nociceptive pathway, thereby enhancing pain management and minimizing opioid use. Opioid-sparing or opioid-free approaches can be effective for perioperative analgesia; however, their necessity and appropriateness depend on the circumstances of the individual patient. Common perioperative analgesics include opioids and non-opioid systemic analgesics, such as dexmedetomidine (DEX), ketamine, acetaminophen, non-steroidal anti-inflammatory drugs, gabapentinoids, and regional or local anesthetics administered intravenously or through infiltration.

Among these agents, DEX has emerged as a noteworthy alternative or adjunct to opioids in perioperative settings. Its immunomodulatory properties distinguish it from the predominant immunosuppressive effects associated with opioids, particularly morphine. Preclinical studies have suggested that DEX exhibits reduced immunosuppressive effects and may provide beneficial immunomodulatory outcomes. It is a superior option when a robust immune response is critical, such as during cancer surgery or other physiological stressors. DEX influences innate immune function through various pathways, including direct action on alpha-2 adrenergic receptors located on immune cells and sympatholytic effects that modulate catecholamine levels and immune cell signaling.^1^ In particular, DEX enhances natural killer (NK) cell function, promotes neutrophil activities such as chemotaxis and phagocytosis, and preserves macrophage activation while reducing the secretion of pro-inflammatory cytokines.^2^ DEX exhibits pro-tumorigenic and anticancer effects, depending on the dose. At low doses, it promotes cancer cell survival, proliferation, migration, angiogenesis, and proinflammatory cytokine release via α2-adrenergic receptor activation. At higher doses, DEX induces anticancer effects by upregulating proapoptotic proteins, activating caspases, and promoting ferroptosis. It also reduces cell migration and arrests the cell cycle through cyclin D1 downregulation, partly through epigenetic changes. These findings suggest a dose-dependent role, where low doses support tumor growth and clinically relevant doses trigger anticancer responses. This article explores DEX's potential in perioperative pain management, its immunomodulatory effects, and its dose-dependent impact on cancer progression, while highlighting the need for further clinical research.

## Mechanism of Dexmedetomidine's immune effects

Preclinical studies suggest that DEX modulates immunity through several pathways. One key mechanism underlying this effect involves the direct activation of alpha-2 adrenergic receptors on various immune cells, which is crucial for regulating immune responses.^1^ Additionally, sympatholytic effects of DEX influence the central and peripheral nervous systems, resulting in altered catecholamine release and immune cell function modulation.^2^ DEX enhances innate immune responses by increasing NK cell activity, which is essential for recognizing and destroying tumor and virally infected cells. Moreover, DEX promotes critical neutrophil functions including chemotaxis, phagocytosis, and superoxide radical production, vital for effective immune response to pathogens.^3^ It also supports macrophage activation, enabling antigen presentation and cytokine production, while decreasing pro-inflammatory cytokine secretion, thereby mitigating excessive inflammation.^3^ Nevertheless, additional clinical research is required to validate these mechanisms in human populations and investigate the potential implications of DEX in perioperative pain management and cancer therapy.

## Immune markers

A meta-analysis by Wang et al., in 2019^3^ reviewed 67 studies, including 59 randomized controlled trials (RCTs) and eight cohort studies, involving a total of 4842 patients (2454 in the DEX group and 2388 in the control group). The analysis covered various types of surgeries, such as cardiac, abdominal, thoracic, spine, orthopedic, genitourinary, and cancer surgeries, and compared DEX with placebo, propofol, morphine, hydromorphone, fentanyl, and midazolam. The study found that perioperative DEX administration was associated with reduced levels of epinephrine, norepinephrine, cortisol, glucose, IL-6, TNF-α, and CRP, along with increased IL-10 levels.

Recent RCTs have corroborated the predominant anti-inflammatory effects of DEX.^4^^,^^5^ In a study involving patients undergoing posterior lumbar interbody fusion, the postoperative effects of fentanyl were compared to those of a combination of fentanyl and DEX. A study of posterior lumbar interbody fusion patients found that fentanyl-DEX combination increased T helper 1 (Th1) and regulatory T (Treg) cells, indicating preserved immunity and anti-inflammatory effects.^4^ Another investigation assessed the impact of intraoperative and postoperative DEX *vs.* placebo on NK cell function in patients with uterine cancer, revealing increased interferon-gamma (IFN-γ) levels in the DEX group.^5^ These findings were unexpected, as DEX has previously been linked to decreased IFN-γ. Thus, the researchers hypothesized that these results may be influenced by the tumor environment created by invasive cervical carcinoma. Additionally, a study involving patients without cancer who underwent laparoscopic cholecystectomy demonstrated a dose-dependent inverse relationship between DEX administration and CRP levels, suggesting that the anti-inflammatory effects of DEX may be titratable.^6^

## Immune cells

Wang et al.^3^ reported that DEX is associated with an increased expression or count of NK cells. Similarly, an RCT involving patients with lung cancer demonstrated a higher NK cell count in the flurbiprofen and DEX groups than in the flurbiprofen-only group.^7^ In contrast, patients with uterine cancer showed no significant differences in NK cell activity.^5^ Regarding monocyte function, an RCT of patients classified as American Society of Anesthesiologists (ASA) category 1 or 2 undergoing multilevel spinal fusion indicated that patients receiving DEX exhibited decreased levels of pro-inflammatory cytokines and increased levels of cytokines associated with intact immune function.^8^ However, further research is required to clarify the effects of DEX on monocyte phagocytosis and antigen presentation.

In one meta-analysis^3^ and other RCT studies, perioperative DEX use in patients with oral and colon cancer correlated with an increased Th1:Th2 ratio and a higher CD4+:CD8+ ratio, suggesting a preserved capacity for cell-mediated immune responses.^4^^,^^7^^,^^9^^,^^10^ Additionally, a study by Lee et al.^6^ on healthy patients who underwent laparoscopic cholecystectomy found that higher intraoperative doses of DEX were associated with elevated IFN-γ/IL-4 ratio (surrogate for Th1:Th2 ratio), and higher IL-17/IL-10 ratio (surrogate for Th17:Treg ratio, which can be interpreted as a marker of immune balance similar to Th1:Th2), indicating a potential for titratable immunomodulatory effects. DEX may counteract the immunosuppressive effects of surgical trauma, which are typically associated with a reduced Th1:Th2 ratio. The immunomodulatory effects of DEX may be titrated in a dose-dependent manner. However, the mechanism underlying alpha-2 agonists’ effects on the Th1:Th2 and Th17: Treg ratios remains unclear.

Furthermore, the meta-analysis by Wang et al.^3^ indicated that DEX is associated with increased B cell expression. A more recent study reported no significant difference in B lymphocyte counts between DEX and control groups,^9^ suggesting that, although cellular adaptive immunity may be preserved, there may be minimal or no observable changes in humoral immunity. Additional studies are required to elucidate the effects of DEX on adaptive humoral immunity.

**Figure 1:**
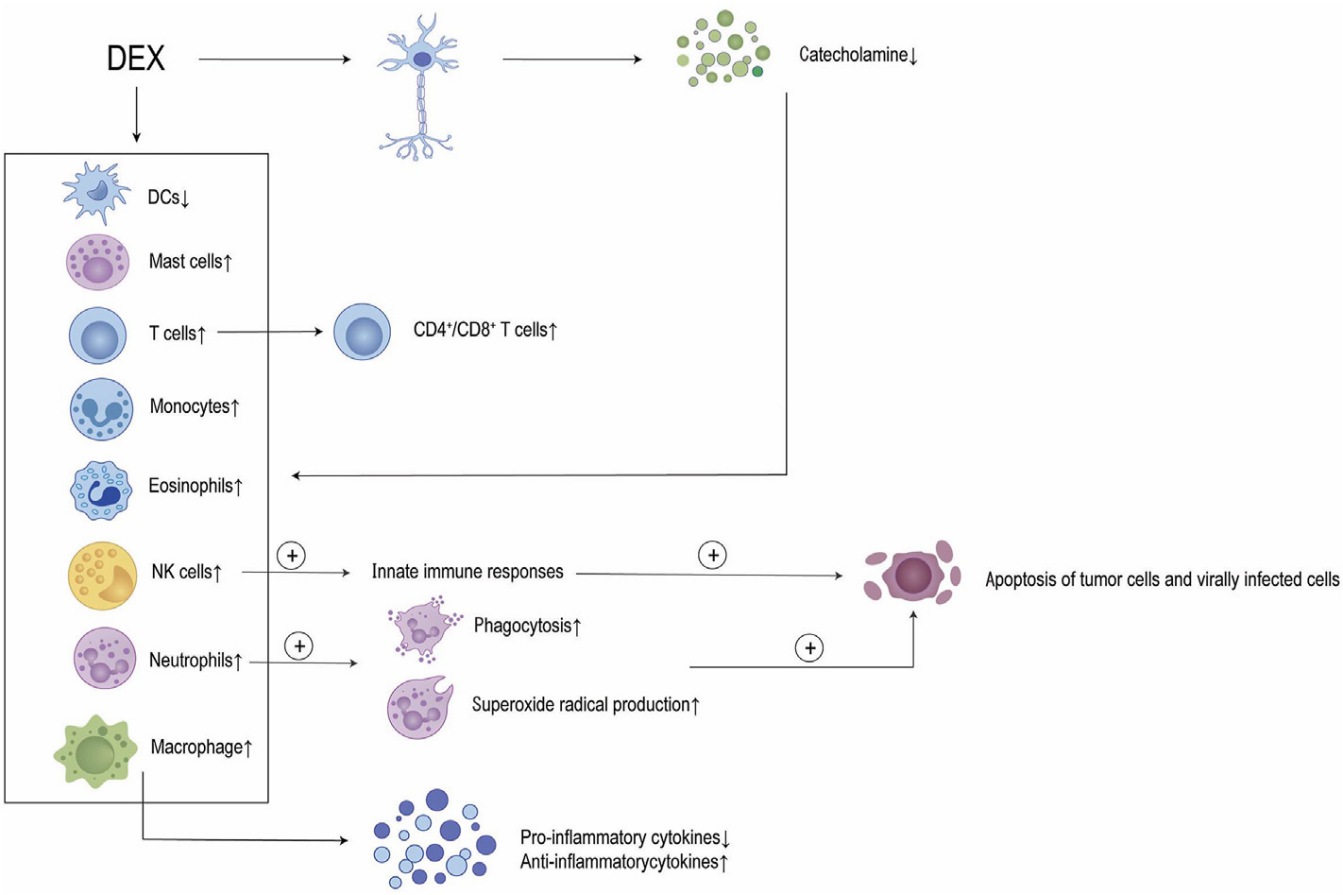
Effect of DEX on immune cells and inflammatory factors. DEX can regulate immune cell function by acting directly on the α2 adrenergic receptors of immune cells or by acting on the nervous system to cause changes in Catecholamine release and regulation of immune cell function, thus suppressing tumor and virally infected cells. CD4^+^/CD8^+^ T cells: Cluster of differentiation 4^+^ helper T cells/cluster of differentiation 8^+^ helper T cells; DC: Dendritic cell; DEX: Dexmedetomidine; NK cells: Natural killer cells.

DEX can regulate immune cell function by acting directly on the α2 adrenergic receptors of immune cells or by acting on the nervous system to cause changes in catecholamine release and immune cell function regulation, thus suppressing tumor and virally infected cells [Figure 1].

## Patient outcomes

Differentiating the immunomodulatory effects of DEX on postoperative outcomes in patients with cancer is challenging because these effects are often intertwined with other actions of DEX such as sympatholysis, analgesia, and anxiolysis. However, DEX has been demonstrated to modulate immune responses in patients with cancer undergoing surgery, influencing both the innate and adaptive immune systems, which may affect postoperative recovery and tumor progression.^11^

Recent studies have examined the potential effects of DEX on cancer progression and postoperative outcomes of different cancer types. A study involving 24 women with breast cancer found that perioperative DEX administration (2 μg/kg) significantly increased the proliferation, migration, and invasion of breast cancer cells *in vitro*, suggesting that DEX may promote tumor progression and potentially impact surgical treatment outcomes.^12^ However, a larger trial investigating the effects of DEX on breast cancer outcomes after surgery (2 μg/kg total dose: 1 μg/kg loading dose, followed by 0.5 µg·kg^−1^·h^−1^ for 2 h) reported no significant differences in recurrence-free survival or overall survival between the DEX and control groups, with immune cell levels similar in both groups.^13^

Similarly, DEX (0.5–1.0 μg/kg) improved liver function by lowering liver enzyme activity and increasing albumin levels in patients with liver cancer. However, it had no impact on inflammation or overall survival, highlighting the drug's potential benefits in supporting liver function postoperatively without altering the disease course.^14^ In colorectal cancer, a follow-up study of 60 patients who received intraoperative DEX (1 μg/kg loading dose, followed by 0.3 µg·kg^−1^·h^−1^ infusion) showed a trend toward improved overall and disease-free survival, although these differences were not statistically significant. The study also revealed no significant impact on mortality or recurrence, although the DEX group had increased rates of vascular and neural invasion, suggesting a potential effect on cancer biology that warrants further investigation.^15^

While studies in non-cancer populations have reported that intraoperative DEX improves outcomes, such as reduced mortality, fewer cardiac and cerebrovascular events, and lower incidence of delirium, the results in patients with cancer are ambiguous. In cardiac surgery, intraoperative DEX has been associated with improved mortality rates and reduced incidences of cardiac and cerebrovascular events and delirium.^16^ Additionally, some studies have suggested that DEX may enhance immune function by regulating cytokine release and promoting the activity of immune cells, such as T cells and NK cells. However, the immunomodulatory effects of DEX in cancer surgery remain underexplored owing to the multifaceted nature of its actions.

Regarding infection rates, sepsis, and other postoperative complications, the effect of DEX on cancer surgery is still under investigation, with mixed results. Some studies have reported a reduction in inflammatory marker levels, suggesting potential immunomodulatory effects.^1^ However, larger and more specific studies are needed to clarify its impact on cancer-specific outcomes, particularly in terms of cancer progression, immune function, and postoperative complications such as sepsis.

Overall, these studies suggest that while DEX may have some benefits in supporting liver function and improving survival trends in certain cancers, its role in tumor progression remains complex and warrants further exploration. More clinical trials are needed to define its therapeutic potential in patients with cancer, particularly with respect to its influence on immune function and postoperative complications. ^1^^,^^10^^,^^16^^,^^17^

## Dexmedetomidine and cancer

Animal and *in vitro* studies have established a potential link between DEX and tumor progression, possibly through the stimulation of alpha-2 adrenergic receptors on tumor cells.^10^ However, clinical studies examining the effects of intraoperative DEX on recurrence-free and overall survival have reported mixed results. Two recent studies, one involving adults undergoing resection for lung adenocarcinoma, and the other focusing on pediatric patients undergoing cytoreductive surgery for peritoneal carcinomatosis, revealed no significant association between intraoperative DEX exposure and recurrence-specific survival or overall survival.^18^ In another propensity-matched trial, although intraoperative DEX did not correlate with recurrence, it was associated with a decrease in the overall survival of patients after surgery for non-small cell lung cancer.^19^ These findings were unexpected as DEX was previously considered potentially advantageous for patients with cancer owing to the lack of immunosuppressive effects associated with opioids and volatile anesthetics, which could contribute to tumor progression.^20^ Thus, current evidence suggests that DEX may not be an ideal multimodal analgesic for patients with cancer, possibly because of differing underlying mechanisms that do not primarily involve immune suppression.

**Figure 2:**
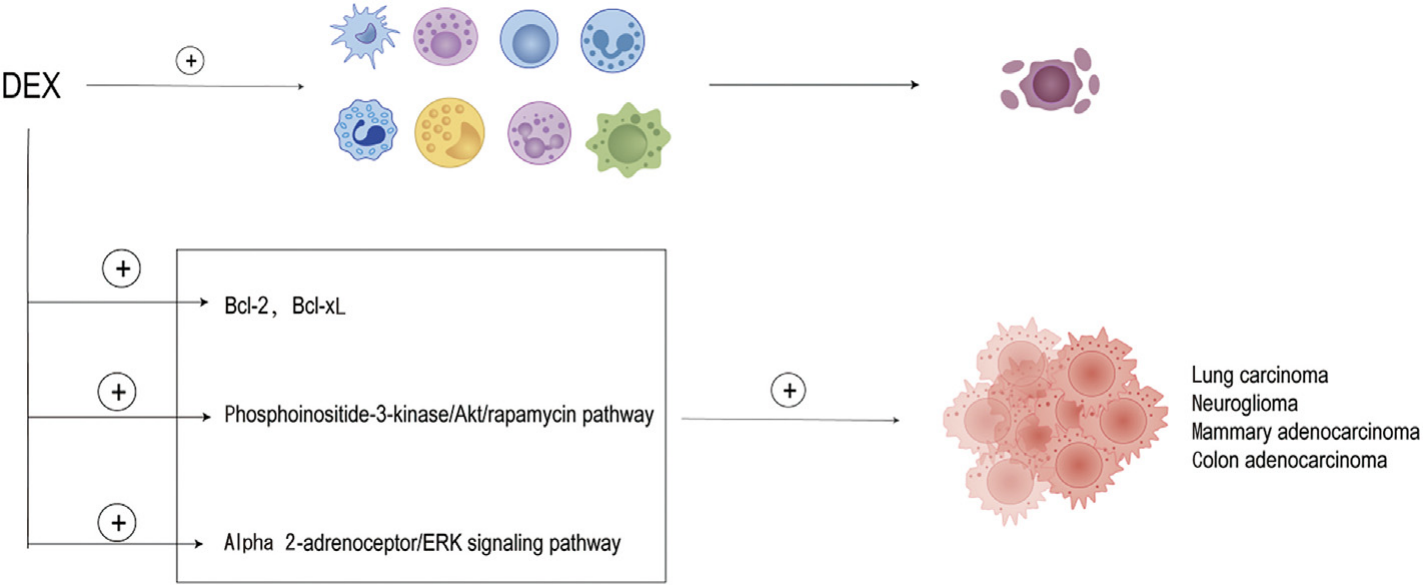
Association between DEX and tumors. DEX inhibits tumor growth by modulating immune cells and inflammatory factors but may promote tumor multiplication by activating relevant signaling pathways through direct action on adrenergic receptors in tumor cells. Akt: Protein kinase B; Bcl-2: B-cell lymphoma-2; Bcl-xL: B-cell lymphoma-extra-large; DEX: Dexmedetomidine; ERK: Extracellular regulated protein kinase; +: Promotion.

DEX inhibits tumor growth by modulating immune cells and inflammatory factors but may promote tumor progression by activating relevant signaling pathways through direct action on adrenergic receptors in tumor cells [Figure 2].

In conclusion, the perioperative use of DEX demonstrates promising immunomodulatory effects, including modulation of the Th1:Th2 and Th17: Treg ratios, which may contribute to improved immune function and reduced inflammation in surgical patients. The findings suggest that DEX may be an effective alternative to opioids for pain management, with potential benefits for patients undergoing cancer surgery or those under significant physiological stress. Further clinical research is needed to clarify the optimal dosing strategies and to better understand the mechanisms through which DEX influences immune responses and cancer progression. These insights could inform more personalized approaches to perioperative pain management.

## Authors contribution

Yuxian Liu: literature review, data curation, writing- original draft preparation; Isabelle Yang: writing- original draft preparation; Xintong Wang: software; He Ma: visualization, validation, supervision; Jingping Wang: conceptualization, validation, supervision and writing – review & editing.

## Ethics statement

None.

## Declaration of generative AI and AI-assisted technologies in the writing process

The authors declare that no generative AI or AI-assisted technologies were used in the creation or writing of this manuscript. All content, including text, data analysis, and figures, was developed independently by the authors.

## Funding

None.

## Data availability statement

The data supporting the findings of this study are available within the article or upon reasonable request from the corresponding author.

## Conflict of interest

The authors declare that they have no known competing financial interests or personal relationships that could have appeared to influence the work reported in this paper.
